# RiceMetaSys: Drought-miR, a one-stop solution for drought responsive miRNAs-mRNA module in rice

**DOI:** 10.1093/database/baae076

**Published:** 2024-08-21

**Authors:** Deepesh Kumar, SureshKumar Venkadesan, Ratna Prabha, Shbana Begam, Bipratip Dutta, Dwijesh C Mishra, K K Chaturvedi, Girish Kumar Jha, Amolkumar U Solanke, Amitha Mithra Sevanthi

**Affiliations:** ICAR-National Institute for Plant Biotechnology, Pusa Campus, New Delhi 110012, India; The Graduate School, ICAR-Indian Agricultural Research Institute, Pusa Campus, New Delhi 110012, India; ICAR-National Institute for Plant Biotechnology, Pusa Campus, New Delhi 110012, India; AKMU, ICAR-Indian Agricultural Research Institute, Pusa Campus, New Delhi 110012, India; ICAR-National Institute for Plant Biotechnology, Pusa Campus, New Delhi 110012, India; ICAR-National Institute for Plant Biotechnology, Pusa Campus, New Delhi 110012, India; The Graduate School, ICAR-Indian Agricultural Research Institute, Pusa Campus, New Delhi 110012, India; ICAR-Indian Agricultural Statistics Research Institute, Pusa Campus, New Delhi 110012, India; ICAR-Indian Agricultural Statistics Research Institute, Pusa Campus, New Delhi 110012, India; ICAR-Indian Agricultural Statistics Research Institute, Pusa Campus, New Delhi 110012, India; ICAR-National Institute for Plant Biotechnology, Pusa Campus, New Delhi 110012, India; ICAR-National Institute for Plant Biotechnology, Pusa Campus, New Delhi 110012, India

## Abstract

MicroRNAs are key players involved in stress responses in plants and reports are available on the role of miRNAs in drought stress response in rice. This work reports the development of a database, RiceMetaSys: Drought-miR, based on the meta-analysis of publicly available sRNA datasets. From 28 drought stress-specific sRNA datasets, we identified 216 drought-responsive miRNAs (DRMs). The major features of the database include genotype-, tissue- and miRNA ID-specific search options and comparison of genotypes to identify common miRNAs. Co-localization of the DRMs with the known quantitative trait loci (QTLs), i.e., meta-QTL regions governing drought tolerance in rice pertaining to different drought adaptive traits, narrowed down this to 37 promising DRMs. To identify the high confidence target genes of DRMs under drought stress, degradome datasets and web resource on drought-responsive genes (RiceMetaSys: DRG) were used. Out of the 216 unique DRMs, only 193 had targets with high stringent parameters. Out of the 1081 target genes identified by Degradome datasets, 730 showed differential expression under drought stress in at least one accession. To retrieve complete information on the target genes, the database has been linked with RiceMetaSys: DRG. Further, we updated the RiceMetaSys: DRGv1 developed earlier with the addition of DRGs identified from RNA-seq datasets from five rice genotypes. We also identified 759 putative novel miRNAs and their target genes employing stringent criteria. Novel miRNA search has all the search options of known miRNAs and additionally, it gives information on their *in silico* validation features. Simple sequence repeat markers for both the miRNAs and their target genes have also been designed and made available in the database. Network analysis of the target genes identified 60 hub genes which primarily act through abscisic acid pathway and jasmonic acid pathway. Co-localization of the hub genes with the meta-QTL regions governing drought tolerance narrowed down this to 16 most promising DRGs.

**Database URL**: http://14.139.229.201/RiceMetaSys_miRNA

Updated database of RiceMetaSys URL: http://14.139.229.201/RiceMetaSysA/Drought/

## Introduction

Rice (*Oryza sativa* L) is a staple food crop for more than half of the global population. During its lifecycle, rice crop faces several biotic and abiotic stress that adversely affect the homeostasis of plants, among which drought remains the major one leading to substantial yield penalty of 50% or even more [1, 2]. Considering the global climate change and uneven rainfall in the recent years and increasing demand for supply of rice, there is immense pressure on the growers as well as the crop improvement fraternity/scientists to enhance rice production even under suboptimal growth conditions [2, 3].

Plants exploit several inherent mechanisms to cope with the drought incidences, which include morphological, physiological, biochemical, and molecular changes in plants that enable them to survive the stress conditions [4, 5]. In rice, several genes have been cloned, characterized, and shown to enhance drought tolerance of rice up to a certain limit but not to that significant level that could be exploited in enhancing the yield penalty at the field level. However, drought-stress-tolerant Quantitative Trait Locus (QTLs) identified from popular drought-tolerant donors and exploited in the breeding programmes for crop improvement have proved to be a huge success at the field level globally [3, 6, 7]. Hence, the molecular basis of drought tolerance is still being explored in a more comprehensive way using advanced genomics and bioinformatics tools developed for data generation and big data analysis.

The secondary regulatory elements such as miRNAs, miPEP, lncRNA, PhasiRNA, TasiRNA, etc. have been shown to modulate gene expression widely under stress conditions [3, 8] and these could be an important missing link in the molecular basis of drought tolerance. Of these various forms of RNAs, miRNAs have been explored more thoroughly under drought stress tolerance in rice by various workers [9–13]. The first study on miRNAs in rice was through the microarray technique and Northern blotting [14], later on with the advancement of next generation sequencing (NGS) techniques led to the sRNA sequencing. Drought-responsive miRNAs in Nagina 22 (N22), IR64, Vandana, IR20, Sahbhagi Dhan, Swarna, KMJ 1-12-3, Pusa Basmati-1, Nipponbare, Aday Sel, IRAT109, ARC-10372, wild rice (*O. rufipogon*, DXWR) at vegetative stage as well as at reproductive stage (reviewed in 3,15) have so far been reported in rice. Some modules such as that of miR156 on SPL genes, miR159 on abscisic acid pathway, specifically MYB genes and miR167 on auxin signalling genes, have also been demonstrated to play a role in drought stress tolerance of rice [9]. However, all these information relating to drought specific miRNAs and target genes are currently scattered in rice which need to be placed in a single platform by utilizing the meta-analysis approach.

The sequence read archives (SRA) database provides an opportunity to the scientific community to access all the RNA-seq and small RNA-seq datasets which in turn allows mining them to identify putative miRNA/mRNA modules for the trait of interest by meta-analysis approach. There is also a mega database for miRNA, miRbase (Version 22.1), which includes 271 organisms (all eukaryotes), 38 589 hairpin precursors, and 48 860 mature microRNAs [16]. There are several plant-specific miRNA databases available that are enlisted in Table 1. From the literature survey we could find only one database named ARMOUR (https://armour.icgeb.trieste.it/login; 17) which is rice-specific and includes datasets from salt and heat stress but not accessible due to lack of maintenance.

**Table 1.** Details of the databases developed for various types RNAs in plants

Database name	Summary	link	Reference	Accessibility status
SoMART	Webserver for identification and analysis of miRNAs or tasiRNAs that were involved in the regulation of target genes	http://somart.ist.berkeley.edu	[54]	No
PASmiR	Manually curated database for miRNAs and their targets genes expressing under abiotic stress in over 30 plant species	http://pcsb.ahau.edu.cn:8080/PASmiR http://hi.ustc.edu.cn:8080/PASmiR	[55]	No
PMTED	Expression ATLAS for plant-specific miRNAs target genes by use of microarray datasets	http://pmted.agrinome.org	[56]	No
PNRD	Comprehensive database of all kind of non-coding RNA from ∼150 plant species with their target genes from publically available datasets	http://structuralbiology.cau.edu.cn/PNRD/index.php	[57]	Yes
PmiRExAt	Database for wheat, rice and maize containing the miRNA information and their tissue wise expression profiles	http://pmirexat.nabi.res.in	[58]	No
PmiREN	Database containing comprehensive information about miRNA and its expression in various tissue, target genes and their regulatory networks in plants from large datasets	https://www.pmiren.com	[18, 19]	Yes
TarDB	Database of miRNA target genes across the species and their conservation	http://www.biosequencing.cn/TarDB/	[59]	Yes

Considering the importance of the role of miRNAs in drought stress in rice and the lack of comprehensive platform to access the information for further utilization, here, we present a database named RiceMetaSys-Drought-miR, which has been exclusively developed for drought-responsive miRNAs in rice and their possible target genes with the following objectives: (i) to create a one-stop solution for drought-stress-related miRNAs in rice by which user can compare the expression for particular miRNAs under drought stress from various genotypes (drought tolerant and drought stress sensitive) as well as at different developmental stages, (ii) to identify high confidence target genes of drought-responsive miRNAs from degradome datasets to minimize the prediction of false-positive targets, (iii) to confirm the effect of drought-responsive miRNAs by linking them with the drought-specific expression of the predicted target genes using an updated database on drought-responsive genes in rice (RiceMetaSys: DRGs), (iv) to catalogue rice-specific novel miRNAs, and (v) to design and develop Simple Sequence Repeat (SSR) markers based on drought-specific miRNAs.

We further subjected the target genes of the drought-responsive miRNAs, identified in this report, to network analysis and co-localized them in the drought-responsive meta-QTL regions and further validated those genes by *in silic*o expression analysis.

## Material and methods

### Data resources and mining

For data collection, we thoroughly searched the Google Scholar, PubMed, NCBI, etc. with the keywords such as miRNA, drought, rice, RNA-seq, and retrieved the information about drought-stress-specific sRNA-sequencing data of rice which were publicly available. In this study, we included 28 sRNA datasets from eight widely grown rice genotype, viz N22, IR64, Vandana, Aday Sel, Japonica, Swarna, IR20, Sahbaghi Dhan from different tissue about which detailed information is given in Supplementary Table S1A. Further, we added 30 degradome datasets of rice from different developmental stages and tissue (not exclusively drought specific since the idea was to ascertain the miRNA-mRNA module) for target prediction to the database so that the target prediction quality could be ascertained by the user. (3,18,19: https://www.pmiren.com/browsehref4?wzid=204&type=Degradome; Supplementary Table S1B).

### sRNA analysis and identification of known and novel miRNAs

Sequencing data were initially subjected to quality check using fastqc [20], and after that data were preprocessed using Trim_galore [21] with the following settings: (i) sequence without adapters were discarded, (ii) low-quality reads (below 30) were removed after adapter removal, and (iii) reads with length of <17 and >35 nucleotides were removed. Clean and high-quality reads were used as input in the miRDeep2 ‘version 0.1.3, released in 2019’ [22] and ShortStack [23] tools. Novel miRNAs were selected on the basis of criteria described by [24, 25] and in brief it includes: (i) mature miRNA length should not be >24 and <20 nucleotide, (ii) precursor length should not be <60 nucleotide, (iii) minimum free energy of hairpin should be less than or equal to −15 kcal/mol, (iv) miRDeep score should be >0 and its Rand fold *P* value should be <.05, and (v) it should form hairpin structure when subjected to folding from the RNAFold software from the Vienna package. Apart from this, strict parameters were applied to the novel miRNAs, for them to be considered as true novel miRNA. Each one of them was subjected to psRNA Target [26] software for target prediction with strict parameter such as Expectation ‘2’, Inhibition range ‘9-11’ and only those targets that satisfied these criteria were considered as novel miRNA and used for further analysis. Novel miRNAs were named according to their distribution on chromosome in ascending order (e.g. OsChr01_NovelmiR001). For identification of differentially expressed known miRNAs, edgeR in R [27] was used for which input is raw reads count. Selection of miRNAs was done on the basis of *P* value (<.05) and expression in at least one genotype/tissue with log2FC >1 or log2FC <−1.

### Target prediction

For target prediction of all the known and novel miRNAs, degradome datasets were utilized to minimize the false-positive prediction of target genes. Raw data from all the degradome were subjected to rigorous pre-processing which include quality check, trimming, and removal of low-quality reads. All raw data were merged in a single file and used for target prediction. For degradome target prediction, we utilized the CleaveLand pipeline [28]. Output of CleaveLand pipeline is described in five categories (0–4), of which we considered only 0, 1, and 2. To reduce the number of target gene and increase the specificity, Allen score <7 was considered. Further, to confirm the nature of drought responsiveness of the predicted target genes, we utilized the drought database RiceMetaSys ‘an inventory of microarray and transcriptome data under drought stress’ [29].

### GO enrichment and pathway analysis

GO enrichment of all the genes matched with RiceMetaSys was done with agriGO [30] and for the pathway analysis we utilized KOBAS-i (http://bioinfo.org/kobas; 31).

### Transcription factor prediction, hub gene identification, colocalization of hub genes in the drought metaQTL, and in silico expression profiling

All the target genes validated by RiceMetaSys were subjected to iTAK (http://itak.feilab.net/cgi-bin/itak/index.cgi; Zheng *et al*. [32] last accessed on 20 October 2023) for the identification of transcription factors. For the hub gene identification, initially, we obtained the network of all the target genes of known miRNAs that matched with RiceMetaSys from RiceFREND (33; last accessed on 20 October 2023) and used this as an input in Cytoscape (version 3.10, 34). All the individual networks were merged while the scattered networks were excluded from further analysis. Preliminary network analysis was performed to check the optimal network properties, such as node degree distribution and clustering coefficient. Top 20 hub genes were identified from cytohubba plugin [35] on the basis of degree and maximal clique centrality (MCC) algorithm and hub genes were also identified by using CentiScape on the basis of centrality [36]. For co-localization of the hub genes in the identified drought QTLs regions, we utilized information of MetaQTL from Khahani *et al*. [37], and the hub genes were searched in the identified MetaQTL region. *In silico* expression profiling of the identified hub genes that were colocalized in the MetaQTL region was done by utilizing the RiceMetaSys-Drought [29] and RiceXpro [38].

### Designing of drought-responsive miRNA SSR markers

All the identified drought-responsive miRNAs were used for SSR marker development. Information about all the miRNA precursor and sequence was retrieved from miRbase (16; last accessed on 20 October 2023). For prediction of SSR markers, 500bp upstream as well as downstream sequences were retrieved from the rice genome by using bedtools [39]. SSR was predicted using MegaSSR [40] and for primer synthesis, di-, tri-, tetra-, penta-, and hexa-SSRs of minimum 12 bp length were chosen. Criteria employed for primer synthesis were: primer length of 18–25bp, melting temperature (Tm) at 50–65°C range, GC content of 40–60%, and product size of 100–300 bp length.

### Updating the RiceMetaSys—Drought RGs

We have previously developed RiceMetaSys: Drought RGs [29] by meta-analysis of microarray dataset under drought stress from various rice genotypes. With the advent of NGS, several studies utilizing transcriptomics approaches are available in rice under drought stress from different genotypes and tissue. Here, we have updated the previous version of this database with RNA-seq data of five rice genotypes, viz N22, IR64, Chandan, Pokkali, and Tempha under drought stress. This included five tissues (flag leaf, panicle, root, inflorescence, and seedling) covering vegetative as well as reproductive stage [41–44]. We obtained the differential expression gene list from the original papers and filtered them on the basis of *P* value <.05 and log2 >2 or log2<−2. In addition to that, SSR markers for the newly added genes were designed using MegaSSR [40].

### Database construction and design

The drought responsive genes (DRG) part of the earlier RiceMetaSys database comprised of drought microarray-based datasets [29]. In the present study, this is updated with drought-specific transcriptome datasets [41–44]. The updated database is accessible from the following URL: http://14.139.229.201/RiceMetaSysA. The front end of the database was developed with HTML5 and CSS. All the datasets were stored in the MariaDB relational database system. Chart.js is employed in the database to show the expression graphically. Data Tables were also added for the advanced interaction, and downloads in Excel format were enabled. Further, PHP is the server-side scripting language employed in the database. The database is hosted in the Linux operating system using the XAMPP (Apache, MariaDB, PHP and Perl) server. The hmmer tool [45] was used to search the user-provided sequence in the database. The miRNA target genes were hyperlinked with the RiceMetaSysA: DRG Database.

## Results

### Statistics of RiceMetaSys: drought-miR

**Figure 1.**
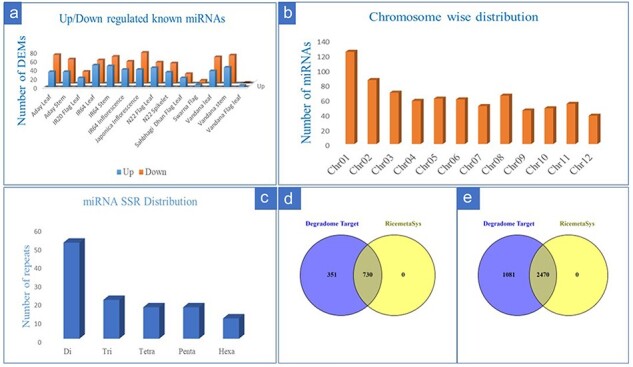
Details of drought-responsive miRNAs in rice identified from multiple miRNA-omics studies. (a) The genotype-wise distribution of upregulated and downregulated known miRNAs under drought stress in rice; (b) chromosome-wise distribution of novel miRNAs in rice; (c) the repeat distribution of SSR specific to drought-responsive known miRNAs in rice; (d and e) evaluation of the target genes (of the known (d) drought responsive and novel (e) miRNAs) in RiceMetaSys database for their drought responsiveness.

From the 28 individual datasets under control and drought stress in rice, we identified a total of 258 known miRNA by using miRDeep2 (133) and ShortStack (196). A greater number of miRNAs was detected by ShortStack as compared to miRDeep2 while common miRNA between the two tools was only 27.5% (Supplementary Figure S1). From 258 detected miRNAs, we identified a total of 216 differentially expressed known miRNAs at least in one genotype. Out of these, the number of upregulated miRNAs in at least one genotype corresponded to 158 whereas downregulated miRNAs were 187 suggesting differential expression of miRNAs under drought in different genetic backgrounds. Genotype-wise comparison for number of differentially expressed miRNAs (DEMs) also revealed that the number of downregulated miRNAs was more as compared to upregulated miRNAs (Fig. 1a). The DEMs that were represented by more than three genotypes/tissue include members from the Osa-miR156, Osa-miR 160, Osa-miR166, Osa-miR167, Osa-miR396, Osa-miR408, and Osa-miR530 families. From 8 genotypes, 5 tissues, and 28 datasets, we identified 759 putative novel miRNAs that satisfied the strict criteria laid down and described in the material and method section. Chromosome-wise distribution of identified novel miRNAs revealed that most of the novel miRNAs were on Chromosome 1 (Fig. 1b). For the identification of drought-responsive miRNA-based SSR markers, we initially considered all the 216 identified drought-responsive miRNAs; however, after removal of redundancy (i.e. miRNAs having same hairpin structure for variant of miRNAs), only 161 sequences were available for SSR mining. From these, the maximum number of repeats identified was for di-repeat followed by tri, tetra, penta, and hexa repeats (Fig. 1c). Finally, SSR primers were designed for 54 drought-responsive and known miRNAs, after the removal of mono repeats. Target prediction from 30 degradome datasets for known miRNAs resulted in 1558 target genes for 193 miRNAs out of which 1081 were unique entries. Target genes prediction of the remaining 23 DEMs did not yield any results after applying filter on the predicted target genes (only 0–2 categories were considered). Comparison of the target genes predicted for the known miRNAs with RiceMetaSys (repository on drought-stress-responsive genes) revealed that out of the 1081 unique genes, 730 genes (67.62%) differentially expressed under drought conditions in at least one genotype (Fig. 1d). Target prediction of 759 putative novel miRNAs resulted in 5680 target genes for 505 novel miRNAs, of which the unique target genes amounted to 3552. From these, 2468 genes (69.52%) were found to match with drought-responsive gene catalogue of RiceMetaSys (Fig. 1e).

### Database construction

**Figure 2.**
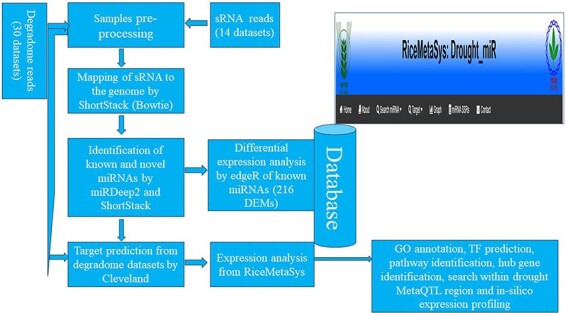
The workflow adopted for the construction of the relation database RiceMetaSys.

Results obtained from the *in silico* analysis were further used for the development of a web-interface/database (Fig. 2). All the data related to the drought-specific known miRNAs such as expression of miRNAs across tissues and genotypes, target genes of miRNAs, and that of novel miRNAs such as their mature miRNA details, precursor sequences, secondary structures and their information, target gene details including degradome peak were stored in MariaDB relational database system. The database is hosted in the Linux operating system using the XAMPP (Apache, MariaDB, PHP, and Perl) server. The RiceMetaSys: Drought-miR database is accessible from http://14.139.229.201/RiceMetaSys_miRNA.

### Important features of the database

**Figure 3.**
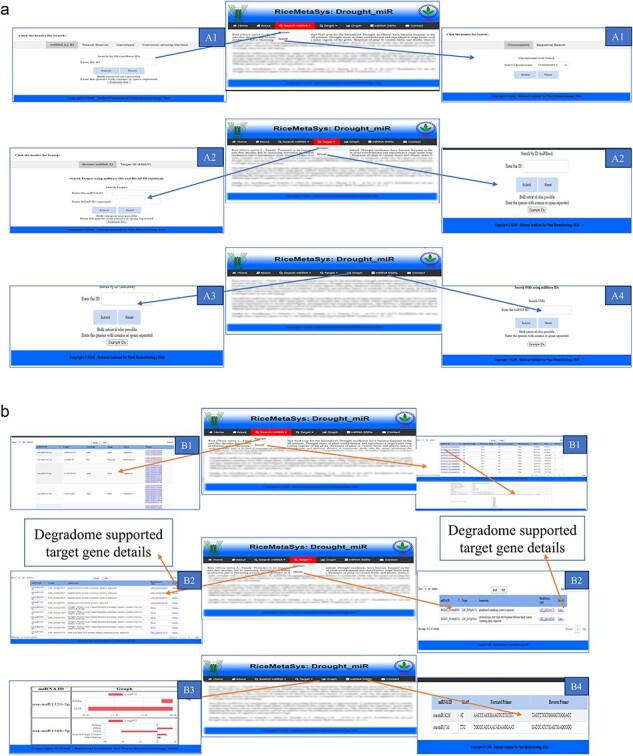
Snapshots of the database, RiceMetaSys: Drought_miR, demonstrating its utility in terms of search functionalities and the nature of outputs generated: A1: input search options (miRbase id, tissue, genotype, common among varieties, chromosome, sequence) for known and novel drought-stress-responsive miRNAs (DRMs); A2: input search options (miRbase id, target id, chromosome-wise) of target genes of DRMs; A3: graph for comparative expression of DRMs; A4: retrieval of SSRs from known DRMs B1: output for known and novel DRM search; B2: output for target genes of DRM search; B3; output for comparative expression profiling of DRMs: B4; output of SSR search of DRM.

To identify drought-responsive miRNAs and their target genes reliably, well-analysed and designed, trait-specific, freely accessible, and user-friendly web interfaces could be of service to researchers. They can facilitate better understanding and assist in deducing meaningful inferences from the researchers’ own data or metadata. For this, a separate tab and links have been designed in this database where user can search known miRNAs either by miRbase id, or by specific genotypes, or tissue wise. To understand the miRNAs whether they have one or more type of regulation between genotypes, an option named ‘common miRNAs among the genotypes’ has been enabled. Output of these searches have been designed to give information about miRNA expression (under drought in comparison to control conditions), fold change, tissue and developmental stage sampled, as well as their corresponding target genes identified from the degradome datasets. There is a separate tab for novel miRNAs where users can search for novel miRNA on the basis of their chromosome-wise distribution; in addition to that, there is a provision for querying their own sequences (either mature or hairpin sequence) which will provide information about the best matching novel miRNAs in the database with the details of miRNAs such as their hairpin secondary structure, minimum free energy (MFE), mature and hairpin sequence, genomic coordinates. All the information about known and novel miRNA could be downloaded in excel or pdf format (Fig. 3a and b).

Since miRNAs act through transcripts of the target genes, there is a separate tab for target genes of known and novel miRNAs which has provisions for search in two ways, i.e. either by miRNA id of known or novel miRNAs or by giving the target genes id (Rice Genome Annotation Project ID, MSU7). In addition to that, for novel miRNAs, users can search target genes on the basis of chromosome-wise miRNA distribution. The output page of both the known and novel miRNAs shows the information about miRNAs, their target genes (LOC IDs), and annotation of the target genes. Upon clicking on the specific target gene, the hyperlinked data on the details of target gene and degradome parameters such as degradome category, *P* value, degradome peak, slicing position, minimum free energy, etc. can be obtained. To have meaningful information of the target genes, it is imperative to link their expression data under drought stress. Hence, the drought responsive genes database, RiceMetaSys-Drought RGs, is linked with the miRNA database. This gives the user a complete insight into the expression pattern of the miRNA as well as their targeted mRNAs in a specific genotype/tissue of interest under drought stress. There is an option of graphical representation, enabled for comparing the expression of any known miRNAs among the genotype, and the resulting 2D-graph can be saved in png format. Considering the importance of SSR markers in molecular breeding, miRNA-based SSR markers were designed, and added to the database, by linking them with the respective miRNAs (Fig. 3a and b).

### Gene ontology and pathway analysis

**Figure 4.**
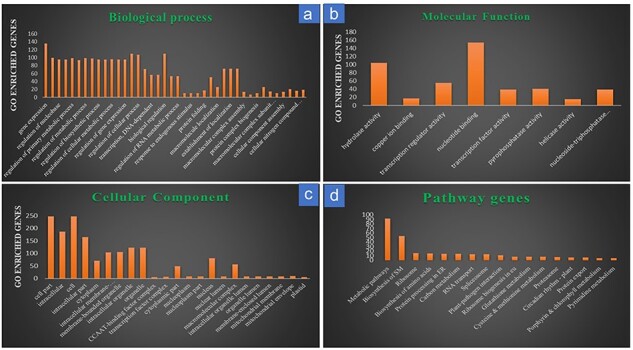
Gene ontology and pathway analysis of the drought-responsive target genes of the drought-responsive known miRNAs. (a) Target gene distribution in the sub-components of biological processes; (b) target gene distribution in the sub-components of molecular functions; (c) target gene distribution in the sub-components of cellular components; (d) target gene distribution across major pathways.

Gene ontology analysis of the 728 target genes of the known DEMs, which matched with RiceMetaSys: Drought RGs, categorized them into three categories, viz., biological process, cellular components, and molecular function. Major sub-components of biological function included gene expression, regulation of metabolic process, regulation of biosynthetic process, etc.; similarly, major cellular component category included intracellular, cytoplasmic, membrane bound organelle genes, etc., while molecular function category included genes that were involved in hydrolase activity, nucleotide binding, regulation of transcription, etc. (Fig. 4a–c). Pathway analysis from Kobas-i revealed that out of the 728 genes, 171 were enriched in various pathways. Maximum genes were involved in biosynthesis and metabolism of biomolecules followed by biosynthesis of secondary metabolites (Fig. 4d; Supplementary Table S2).

### Transcription factor prediction, hub gene identification, and co-localization of the hub genes in the drought meta-QTL

From transcription factor prediction analysis, we found that out of the 728 target genes for known drought-responsive miRNAs, 128 genes belonged to different classes of transcription factors, the major ones being SBP, NAC, ARF, GRF, B3-ARF transcription factor families (Supplementary Table S3). The input for hub gene identification included the same set of 728 drought-responsive genes, for which a network was obtained from RiceFREND and analysed from cystoscope. Based on the MCC criteria and degree criteria, the hub genes were shortlisted. From each of the methods, the top 20 genes were selected. In addition to that, we used CentiScape for identification of inter-modulator on the basis of degree centrality and selected top 20 genes with highest degree centrality. The 60 genes, thus selected, were used in further analysis to ascertain whether they have a prominent role in imparting drought stress tolerance. Out of the 60 hub genes, only 47 could be converted into MSU id from new genome annotation version 7.

Meta-QTLs are the genomic region which are identified by combining consensus loci from different QTLs studies for a single trait or related traits into single dataset which results in the identification of the most accurate position and confidence interval (CI) of the QTL regions. Meta-QTLs are advantageous over a genotype or a study-specific-QTLs as the former are better in terms of increased CI for identification of candidate genes, developing markers, and identification of most consistent QTLs irrespective of genetic background, mapping population and field trial conditions [37]. Hence, we searched the 60 hub genes, identified through three different algorithms, in the 61 Meta-QTL regions governing drought stress tolerance in rice that were earlier identified by combining 563 QTLs from 67 mapping populations from studies published between 2001 and 2019 [37].

**Figure 5.**
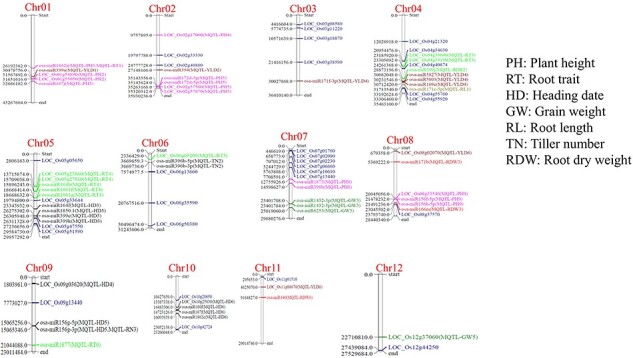
Chromosomal distribution of miRNAs and hub genes identified by network analysis of drought-responsive genes of DRMs and genes co-localized in the drought-responsive meta-QTL region.

Co-localization of differentially expressed miRNAs in the Meta-QTL regions revealed that out of the 216 miRNAs, 37 were co-localized in MQTL for different component traits of drought tolerance (Fig. 5). Out of the 60 hub genes, 16 genes were found to be co-localized in the Meta-QTL region (Fig. 5), which included genes for trait like root thickness [LOC_Os04g39040 (UBA and UBX domain-containing protein), LOC_Os04g39190 (Cell division protease ftsH homolog 4), LOC_Os05g23860 (Rab GDP dissociation inhibitor alpha), LOC_Os05g27010 (Peptide transporter PTR3-A), and LOC_Os06g05209 (Pectate lyase precursor)]; yield [LOC_Os08g02070 (OsMADS26—MADS-box family gene with MIKCc type-box) and LOC_Os11g08670 (NAD kinase)]; plant height [LOC_Os01g54890 (Ethylene-responsive transcription factor 2), LOC_Os01g55050 (Protein of unknown function DUF1421 domain containing protein), LOC_Os02g17060 (OsSub15—Putative Subtilisin homologue), LOC_Os02g57560 (Tyrosine protein kinase domain containing protein), LOC_Os02g57670 (Ribosomal L9), and LOC_Os08g33540 (ATP-dependent Clp protease adaptor protein ClpS containing protein)]; and heading date [LOC_Os09g03620 (Wall-associated receptor kinase-like 20 precursor) and LOC_Os10g25830 (mitochondrial carrier protein)] and for grain weight [LOC_Os12g37060 (Expressed protein)] (Supplementary Table S4). We could find two miRNA-mRNA modules for hub genes that were co-localized in the meta-QTL region which included osa-miR396b-5p/LOC_Os09g03620 (Wall-associated receptor kinase-like 20 precursor) and osa-miR2275d/LOC_Os05g27010 (Peptide transporter PTR3-A).

### In silico expression profiling

**Figure 6.**
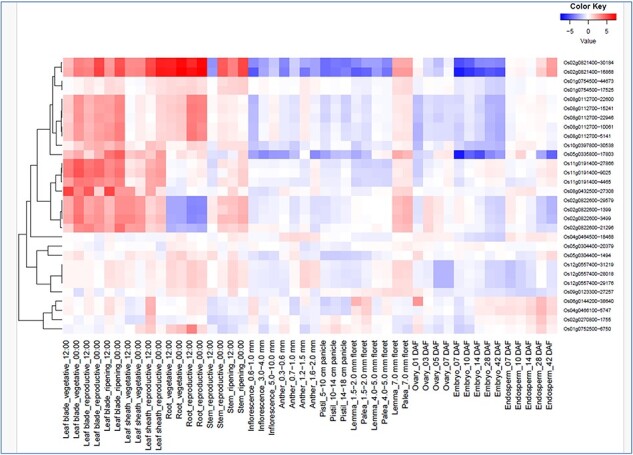
Spatio-temporal gene expression of hub the gene that co-localized in the drought-responsive meta-QTL region in various tissues/organs throughout growth stage in rice under field conditions (from RiceXpro).

*In silico* expression analysis of the 16 hub genes co-localized with the Meta-QTL regions were again checked for their drought responsiveness in RiceMetaSys (inventory of drought responsive genes from large microarray datasets and RNA-seq datasets). RiceMetaSys revealed that out of 16 genes 11 were differentially expressed in at least one genotype. Further, to check the response of these selected genes on the exposure to various hormones, RiceXpro was used and expression profile at different time intervals from shoot and root tissues was obtained (Supplementary Figure S1 A and B). From the expression data, it became evident that these genes were either abscisic acid or jasmonic acid responsive. Further, to confirm the role of these genes, we looked into the expression of these genes at different developmental stages and tissues (Fig. 6) and found that these genes were highly expressed in the leaf blade, leaf sheath, and root in both vegetative and reproductive stages. Thus, the 16 genes narrowed down could be key players in modulating the drought stress response in rice across tissues and developmental stages.

### Updating the RiceMetaSys

Drought RGs- RiceMetaSys previously had 9379 drought-responsive differentially expressed genes from microarray datasets. In the updated version of this database, we have added five more datasets from transcriptomics studies in which Chandan, Pokkali, and Tempha are the new genotypes. From the transcriptomic studies we identified, 14 450 differentially expressed genes at *P* value <.05 and log2 >2 or log2 <−2. Comparison with the genes of existing database revealed that there were 5392 common genes while the remaining 9058 genes were new. Out of these new genes, 5331 were upregulated and 3726 were downregulated under drought stress. In the current version of database, thus, the total number of drought-responsive genes is 18 437, which is almost double from the previous version. For the new unique drought-responsive genes added to this database, we designed SSR markers. The distribution of SSR was highest for tri repeats followed by di, penta, hexa, and tetra repeats (Supplementary Figure S2). From this, we discarded the mono-repeat SSR class and designed SSR primer from the flanking regions of 5256 genes which harboured di to hexa repeat motifs.

## Discussion

Research directed towards understanding sRNAs, especially their role in stress management and development, has immensely increased in the past two decades. This has ultimately resulted in the accumulation of huge amount of sRNA and degradome datasets, necessitating the organization of all that information in a single platform by following uniform criteria of annotation [25]. miRbase is the mega database on miRNA that provides sequence details, annotation, genomic location, tissue-specific expression, and allows browsing from model organisms and major taxa of higher hierarchy. It also has mapped the functional role of miRNA from published literature. miRbase caters to both animal and plant species while PmiREN caters specifically to plant species [16, 18, 19]. A separate database for plants was needed because annotation of plant miRNAs is more complex in comparison with animals which need to follow the updated annotation criteria of miRNA. PmiREN [18, 19] was developed with the aim of identifying plant-specific miRNAs with the updated annotation criteria; this database was developed for identifying the plant-specific miRNAs and their targets were identified using multiomics approach but not for any specific development stage or stress condition. Thus, most of the databases developed so far (Table 1) focused on providing a comprehensive catalogue of miRNAs in the target organisms and their annotation. In rice, ARMOUR database was developed based on miRNAs specific to heat and salinity stress but currently it is not accessible due to lack of maintenance [17]. Developing such trait-specific databases and maintaining them will serve the research community to assign function to the miRNAs and exploit them in genomic selection and genome editing for enhancing trait value of the major crops.

Considering the importance of drought stress in rice and the role of miRNAs in drought stress mitigation and maintaining homeostasis in plants, we developed a one-stop solution for drought-specific miRNAs in the form of ‘RiceMetaSys: Drought-miR’, by following well-defined uniform criteria (for details please see; Material and method section) for the identification of known DEMs as well as novel miRNAs in rice, utilizing all the available miRNA and transcriptome datasets in rice under drought stress. The target gene data have been linked with the degradome datasets for better reliability. Additionally, we linked this database with RiceMetaSys: Drought RGs (Sandhu *et al*. [29]; inventory of drought responsive genes from large datasets) which adds value to the results of miRNA-targeted genes of the DEMs for the user. Since miRNAs, drought-responsive target genes and markers for the DE miRNAs and the DR target genes can be obtained from a single database, we propose this as a unique one-stop solution for all queries on drought-specific miRNAs in rice.

Through meta-analysis of 8 genotypes, represented by 5 tissues and 28 samples from drought stress experiments and following the updated annotation criteria described [3, 25], we identified 759 putative novel miRNAs. Target prediction from degradome datasets of these novel miRNAs identified 5689 target genes, out of which 2468 genes were found to be drought-responsive. This provides high confidence for the authentic annotation of novel miRNAs. We expect this would encourage the researchers to look for the novel miRNAs and their target in their experiments and lead the way for their functional validation.

Since miRNAs work at the secondary level to modulate the expression of target genes and its regulation via QTLs gene have been reported [3], wherein Osa-miR2919 and Osa-miR156k were found to modulate the expression of cytokinin and brassinosteroid pathway genes through the qDTY1.1 region, we explored the metaQTLs identified for drought stress in rice. In this study, we first identified the key genes or hub genes from the DEM targets, by network analysis employing stringent bioinformatics, and co-localized the hub gene in the meta-QTL regions. Our analysis revealed that 16 out of 60 (26.66%) hub genes were found within the MetaQTL regions and from *in silico* expression analysis it became evident that these genes were involved in response to various hormones, especially ABA and jasmonic acid (Supplementary Figure S3A and B). Jasmonic acid has been reported to mitigate the drought stress response by scavenging the excess reactive oxygen species by production of antioxidant enzymes and other protective compounds; in addition to that, it imparts a role in various biotic and abiotic stress by activating the plant defence system [46]. ABA counters the drought response by stimulating the closure of stomata and maintaining water balance [47]. In a similar study on the identification of the hub genes for multiple abiotic stress tolerance in rice, Ramkumar *et al*. [48] reported that 8 of the 17 genes identified by them colocalized with earlier reported QTLs mapped for one or more biotic or abiotic stresses in rice. The miRNA-mRNA modules, osa-miR396b-5p/LOC_Os09g03620 (Wall-associated receptor kinase-like 20 precursor) and osa-miR2275d/LOC_Os05g27010 (Peptide transporter PTR3-A) have been identified in this study by integration of multiomics approach. osa-miR396b has been reported earlier to target the growth-regulating factors transcription factor, which resulted in increase in the salt tolerance [49]. Lin *et al*. [50] reported the role of OsWAK112 (Wall-associated receptor kinase) by overexpression/knockdown in rice as well as in the *Arabidopsis thaliana*, which reveals that it negatively regulates the salt tolerance. From the above finding, we propose that osa-miR396b-5p/LOC_Os09g03620 might be playing an important role in drought stress/osmotic stress. LOC_Os05g27010, Peptide transporter PTR3-A, was recently reported to play a role in transport through OsNPF8.1 in nitrogen uptake and utilization [51]. Overexpression of OsNPF8.1 in rice enhanced drought as well as salinity tolerance and improved the plant growth under low nitrogen conditions. From the above findings, it is evident that the identified miRNA-mRNA modules will be useful to improve the drought resilience in rice and can be a part of the genomic selection directed towards better climate resilience. Our laboratory is working in the multiple aspects of biotic and abiotic stress response of rice and our efforts towards compiling and deciphering the big datasets have resulted in the development of database series of RiceMetaSys for drought, salinity, heat, rice blast disease, and bacterial blight [29, 52, 53]. When these databases were developed, only the microarray datasets specific to a single target stress were considered. Currently, multiple RNA-seq datasets for each of these stresses have been added to the public inventories and such advanced and additional information have necessitated updating of the earlier developed RiceMetaSys: Drought RGs [29]. Hence, we also report the updated database of RiceMetaSys: Drought RGs with 9058 more DRGs which increased the number of DRGs to 18 437 from 9379 genes (http://14.139.229.201/RiceMetaSysA).

## Conclusion

Meta-analysis of drought-responsive sRNA-sequencing datasets and degradome libraries pertaining to different developmental stages led to the identification of 216 DEMs and their corresponding 1081 high confidence target genes. In addition to that, 759 putative novel miRNAs were identified across the datasets and validated through a multi-layered omics approach. Nonetheless, they will need further experimental validation through wet lab experiments. All these results have been accommodated in a user-friendly web interface. Here, we identified drought-responsive hub genes co-localized within the drought-responsive meta-QTLs and their *in silico* expression profiles which revealed their importance in hormonal regulation under drought stress in rice.

## Supplementary Material

baae076_Supp

## Data Availability

Raw reads of sRNA sequencing are available in SRA database and information is given is Supplementary Table S1. All the results have been made available in the web resource.
