# Supplementary material for: RiceMetaSys: Drought-miR, a one-stop solution for drought responsive miRNAs-mRNA module in rice
Source: Database (Oxford). 2024 Aug 21;2024:baae076. doi: 10.1093/database/baae076 (PMC11338179; doi:10.1093/database/baae076)
Supplement: baae076_Supp [file baae076_supp.zip › suppl_data/Supplementary figure.pptx]

## Slide 1
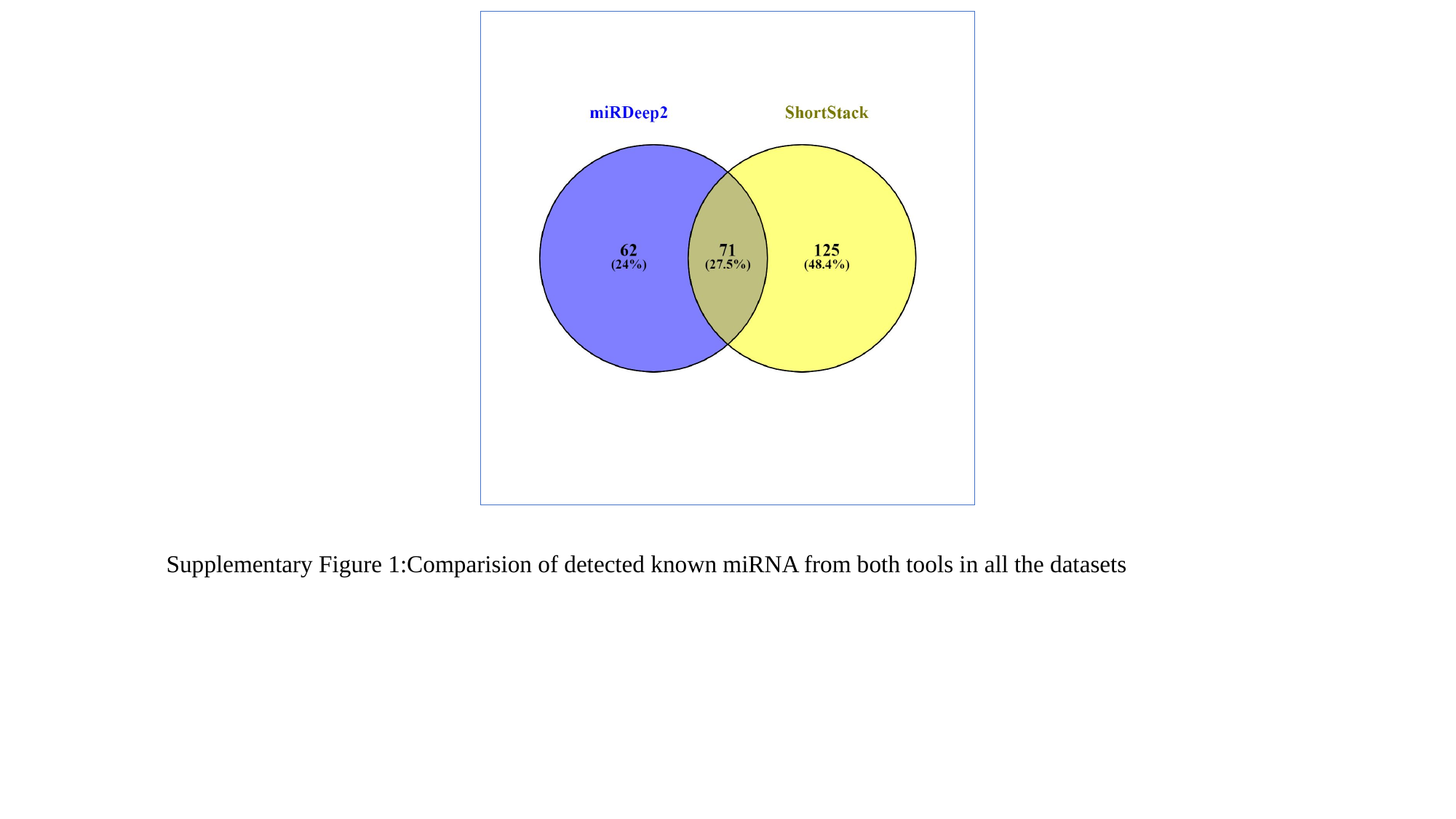

Supplementary Figure 1:Comparision of detected known miRNA from both tools in all the datasets

## Slide 2
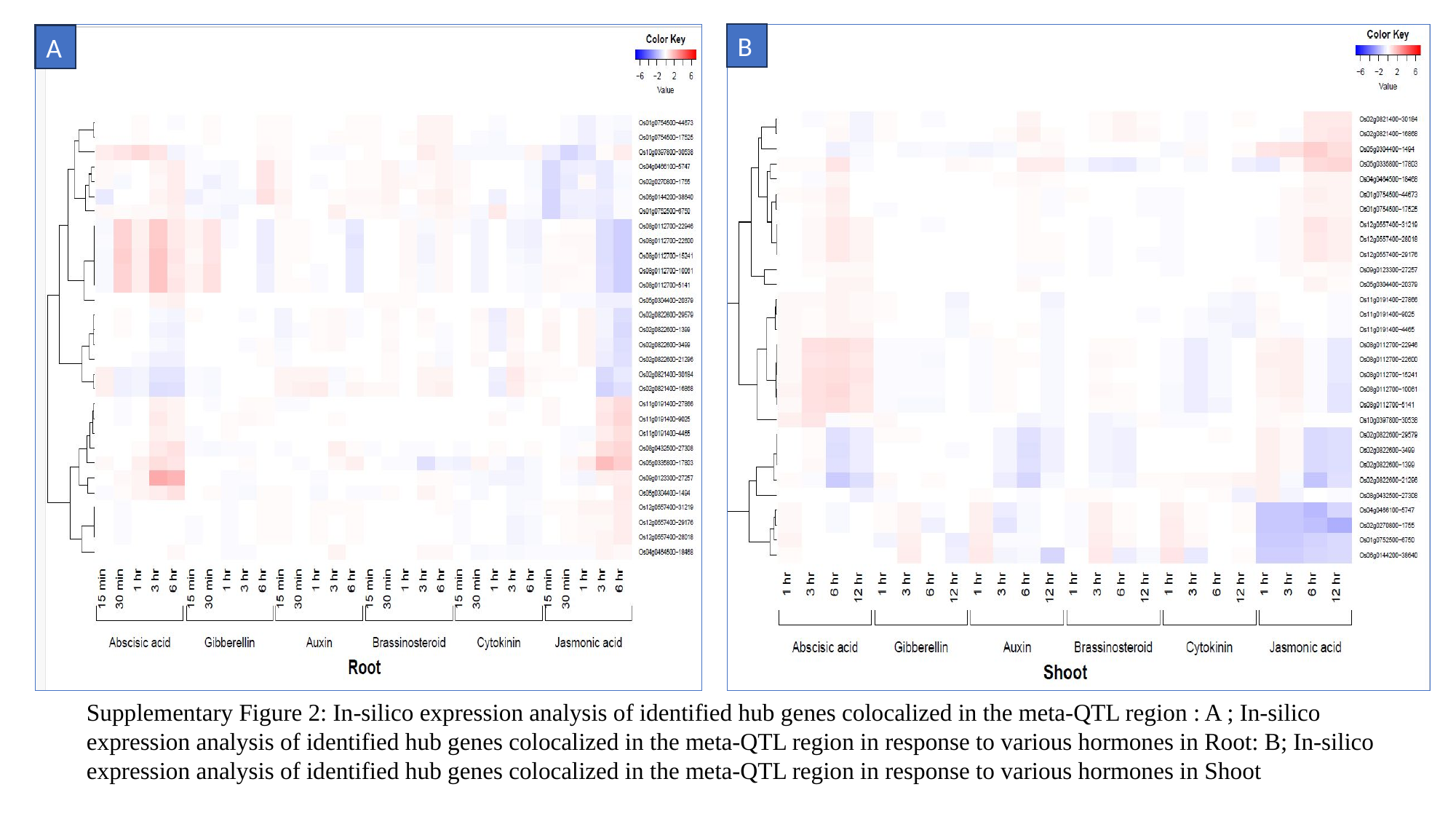

B
A
Supplementary Figure 2: In-silico expression analysis of identified hub genes colocalized in the meta-QTL region : A ; In-silico expression analysis of identified hub genes colocalized in the meta-QTL region in response to various hormones in Root: B; In-silico expression analysis of identified hub genes colocalized in the meta-QTL region in response to various hormones in Shoot

## Slide 3
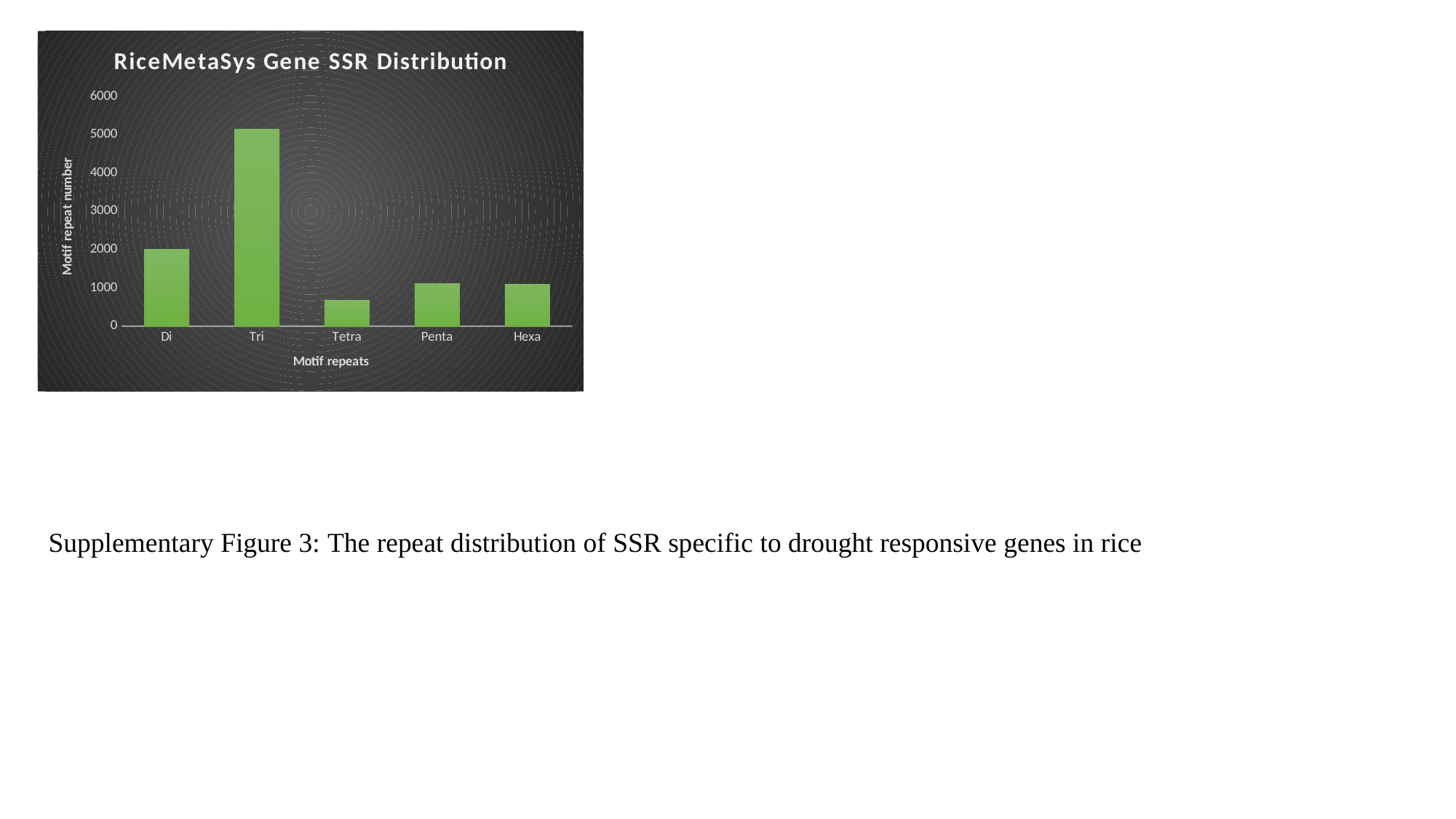

### Chart: RiceMetaSys Gene SSR Distribution
| Category | Total No. of present |
|---|---|
| Di | 2012.0 |
| Tri | 5135.0 |
| Tetra | 677.0 |
| Penta | 1118.0 |
| Hexa | 1095.0 |Supplementary Figure 3: The repeat distribution of SSR specific to drought responsive genes in rice
