# Supplementary material for: RiceMetaSys: Drought-miR, a one-stop solution for drought responsive miRNAs-mRNA module in rice
Source: Database (Oxford). 2024 Aug 21;2024:baae076. doi: 10.1093/database/baae076 (PMC11338179; doi:10.1093/database/baae076)
Supplement: baae076_Supp [file baae076_supp.zip › suppl_data/Supplementary Table 1.docx]

| SRA id | Genotype | Stage | Tissue | Reference |
| --- | --- | --- | --- | --- |
| SRR2458631  SRR2293667 | N22 | Reproductive Stage | Flag leaf | Balyan et al., 2017 |
| SRR2458943  SRR2459139 | N22 | Reproductive Stage | Heading spikelet | Balyan et al., 2017 |
| SRR5556627  SRR5556628 | IR64 | Reproductive Stage | Inflorescence | Cheah et al., 2017 |
| SRR089620  SRR089619 | Japonica group | Reproductive Stage | Inflorescence | Barrera et al., 2012 |
| SRR2097779  SRR2097778 | Vandana | Vegetative Stage | Leaf | Cheah et al., 2015 |
| SRR2098557  SRR2098560 | Vandana | Vegetative Stage | Stem | Cheah et al., 2015 |
| SRR2099567  SRR2099568 | IR64 | Vegetative Stage | Leaf | Cheah et al., 2015 |
| SRR2099745  SRR2099746 | IR64 | Vegetative Stage | Stem | Cheah et al., 2015 |
| SRR2098822  SRR2098823 | Aday Sel | Vegetative Stage | Leaf | Cheah et al., 2015 |
| SRR2099428  SRR2099436 | Aday Sel | Vegetative Stage | Stem | Cheah et al., 2015 |
| SRR19783681  SRR19783682 | Vandana | Reproductive Stage | Flag leaf | Kumar et al., 2023a |
| SRR19783679  SRR19783680 | Sahbaghi Dhan | Reproductive Stage | Flag leaf | Kumar et al., 2023a |
| SRR19783677  SRR19783678 | IR20 | Reproductive Stage | Flag leaf | Kumar et al., 2023a |
| SRR24733501  SRR24733502 | Swarna | Reproductive Stage | Flag leaf | Kumar et al., 2024 |

Supplementary Table 1A: sRNA datasets used for identification of known and novel miRNAs

Supplementary Table 1B: Degradome datasets list used for target prediction of known and novel miRNAs

| SRR032097 | SRR032098 | SRR034102 | SRR039716 | SRR039717 | SRR039718 |
| --- | --- | --- | --- | --- | --- |
| SRR039720 | SRR1609324 | SRR1609325 | SRR1849887 | SRR1849888 | SRR184989 |
| SRR1849891 | SRR1849892 | SRR1849893 | SRR1849894 | SRR1849902 | SRR184993 |
| SRR1849905 | SRR1849906 | SRR1849907 | SRR1849908 | SRR1849909 | SRR184990 |
| SRR3140959 | SRR3140960 | SRR039719 | SRR184980 | SRR184994 | SRR314098 |
